# Strengthening of Existing Bridge Structures for Shear and Bending with Carbon Textile-Reinforced Mortar

**DOI:** 10.3390/ma10091099

**Published:** 2017-09-19

**Authors:** Martin Herbrand, Viviane Adam, Martin Classen, Dominik Kueres, Josef Hegger

**Affiliations:** Institute of Structural Concrete, RWTH Aachen University, 52056 Aachen, Germany; vadam@imb.rwth-aachen.de (V.A.); mclassen@imb.rwth-aachen.de (M.C.); dkueres@imb.rwth-aachen.de (D.K.); jhegger@imb.rwth-aachen.de (J.H.)

**Keywords:** concrete, textile-reinforced mortar, strengthening, shear, bending

## Abstract

Increasing traffic loads and changes in code provisions lead to deficits in shear and flexural capacity of many existing highway bridges. Therefore, a large number of structures are expected to require refurbishment and strengthening in the future. This projection is based on the current condition of many older road bridges. Different strengthening methods for bridges exist to extend their service life, all having specific advantages and disadvantages. By applying a thin layer of carbon textile-reinforced mortar (CTRM) to bridge deck slabs and the webs of pre-stressed concrete bridges, the fatigue and ultimate strength of these members can be increased significantly. The CTRM layer is a combination of a corrosion resistant carbon fiber reinforced polymer (CFRP) fabric and an efficient mortar. In this paper, the strengthening method and the experimental results obtained at RWTH Aachen University are presented.

## 1. Introduction

**Figure 1:**
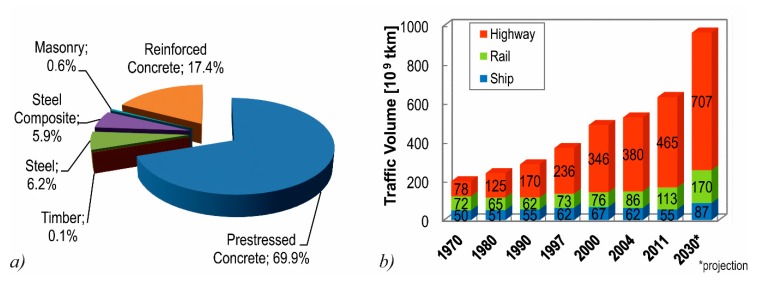
(**a**) German Federal Highway Bridges in terms of bridge deck area [1]; and (**b**) heavy goods traffic volume in Germany [4].

As an essential part for the national infrastructure, the German Federal Highway System comprises about 39,000 bridge structures. In terms of the bridge deck area, almost 90% of these bridges are made of reinforced or pre-stressed concrete [1] (Figure 1a). The traffic volume on these bridges has been increasing over the past decades, particularly concerning heavy goods traffic [2,3], and is expected to further increase in the future [4] (Figure 1b). Especially, abnormal traffic loads and unapproved excessive charges lead to bridges loaded to their design capacity or beyond [5]. 

This results in an accelerated deterioration of these structures, as well as to deficits concerning the integrity according to the applicable code provisions. The aforementioned problems are quite common in western countries [6,7,8,9]. In Germany, the shear design check is critical in most cases [10,11,12]. One reason is that many bridges have been designed according to the so-called principle tensile stress criterion (thus requiring no shear reinforcement). The required minimum shear reinforcement then was less than it is today [13,14]. Although guidelines for the recalculation and assessment of existing bridges have been developed [15,16], and further refinements in shear design models can lead to a significantly higher shear capacity [17,18,19], severe damage often leads to instantaneous need for action, since the damage often has already progressed.

Different strengthening and rehabilitation measures with individual advantages and disadvantages are available to extend the service life of bridges [20]. One of the most common strengthening measures to avoid traffic obstructions is the use of additional post-tensioning in the longitudinal direction [21]. Research has revealed, however, that additional post-tensioning only slightly increases the ultimate shear capacity [14,22]. To provide innovative solutions to the given problems, the use of an additional mortar layer with non-corrosive reinforcement made from carbon fibers is investigated at RWTH Aachen University. This material is called carbon-textile-reinforced mortar (CTRM) and could serve as a conservation method and as a retroactive supplement to the overused mild reinforcement. In the past, the general load-bearing behavior of textile-reinforced concrete (TRC) has been investigated in numerous research projects [23,24,25,26,27]. In the long-term, TRC has the potential of replacing regularly-reinforced concrete in many applications [11,28], e.g., for use in new and filigree structures [29,30,31,32,33,34,35]. The strengthening of existing structures with CTRM or TRC is also currently investigated and has already been successfully applied [36,37,38,39].

The effect of an additional CTRM layer for the webs of pre-stressed concrete beams under cyclic and static loading [40] and for bridge deck slabs [41] is presented within this paper. Previous investigations on RC beams under cyclic loading are described in [42]. The CTRM layer is composed of a carbon fiber reinforced polymer (CFRP) fabric and an efficient mortar. CFRP is corrosion resistant and has a significantly higher tensile strength than rebar steel. Therefore, thin structural components with high strengths can be realized. Reinforced concrete slab segments (*h* = 0.28 m) were strengthened to investigate the effect of an additional CTRM layer on the shear and flexural strength of bridge deck slabs. In addition, the webs of two pre-stressed concrete beams (*l* = 6.5 m, *h* = 0.7 m) were strengthened with CTRM and tested under cyclic and static loading to investigate the effect on the shear strength of the longitudinal system. In this paper, the impact of strengthening layers on the members’ strength is presented.

## 2. CTRM Layer for Bridge Deck Slabs

### 2.1. Concept

**Figure 2:**
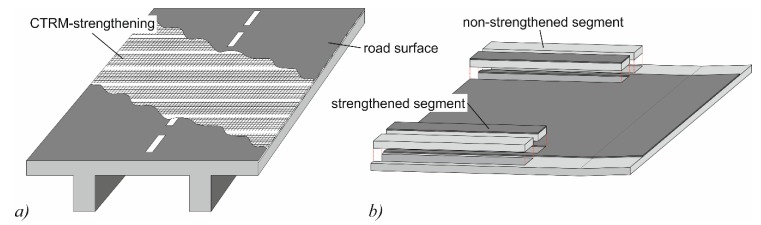
(**a**) Application of Smart-Deck; and (**b**) the position of sawn segments in a demonstrator slab.

With respect to bridge deck slabs, the CTRM layer is called Smart-Deck, as it combines the following three features: all-over real-time humidity monitoring, a preventive cathodic corrosion protection (CCP), and a strengthening effect in the transverse direction of the bridge. Smart-Deck is applied between the surface of the reinforced concrete bridge deck slab and the road surface (Figure 2a). It is installed in segments to obtain defined sectors in the longitudinal direction, which allows for locating possible leaks in the road surface. The monitoring function provides the detection of leaks at an early stage long before severe damage occurs. Due to the possibility of engaging the CCP, the replacement of the damaged road surface can be postponed and realized in convenient periods of low traffic. This allows for an adequate planning horizon and, thus, lower expenses. The bending and strengthening effect of Smart-Deck counteracts the increase in traffic volume and, therefore, extends the remaining service life of the bridge. 

The additional CTRM layer consists of two layers of an epoxy-resin impregnated carbon grid and a high-performance mortar. The carbon reinforcement is equipped with electrical connections for the monitoring and the CCP. The mortar cover is 10 mm thick on both sides, the two reinforcement layers are installed at a distance of 15 mm which makes a total of 35 mm thickness of the additional CTRM layer. Different combinations of mortar and CFRP were tested in an iterative process to meet all requirements of the project. The test specimens presented in this paper were strengthened with a carbon grid with a mesh opening of *e* = 38 mm, a cross-section area of *a*_tex_ = 140 mm²/m and a special epoxy-resin that was complemented with carbon nanotubes (CNT) in order to increase the electrical conductivity (*f*_t,tex_ ≈ 2200 MPa; *E*_tex_ ≈ 215,000 MPa) [43]. In addition, a mortar with a maximum aggregate size of 4 mm that is both flowable and stable enough to gaplessly surround the textile reinforcement was used. The mortar was also required to make for a high conveyor capability, on one hand, and to allow the implementation of an inclination of the surface of at least 2.5%, on the other. The mechanical properties of the mortar were determined on prisms with a length of 160 mm and a width and height of 40 mm. The flexural tensile strength *f*_ct,flex_ amounted to 10.4 MPa and the compressive strength *f*_cm,prism_ to 65.9 MPa, respectively.

### 2.2. Preparation of the Test Specimens

A demonstrator slab was prepared to investigate the implementability under realistic conditions of a construction site on a bridge, the functionality of the monitoring and CCP and the additional strength by Smart-Deck. For this purpose, an approximately 80 m² large RC slab with a height of 0.28 m was built (*f*_c,cyl_ = 58 MPa (*h*_cyl_ = 300 mm; *D*_cyl_ = 150 mm) at time of tests). After four months of curing, the surface was pre-treated with shot peening to increase its roughness for a better bond in the interface between the existing RC structure and the additional CTRM layer. Afterwards, Smart-Deck was applied. In the first step, the carbon grid was secured in place by plastic dowels that were fixed to the RC slab. The mortar was then cast employing a feed hose that was connected to an automatic mixing unit where water was added to dry mortar stored in a silo. The RC slab was reinforced using different longitudinal reinforcement ratios in each third. The respective segments contained 5.24 cm²/m (using bars of Ø = 10 mm every 15 cm), 10.3 cm²/m (Ø14/15), and 25.13 cm²/m (Ø16/8) steel reinforcement (characteristic yield strength *f*_yk_ ≈ 500 MPa). No stirrups or other shear reinforcement elements were used. In order to investigate the strengthening effect of Smart-Deck, two segments were sawn out from the slab in the area of the lowest and highest reinforcement ratio, respectively (Figure 2b). Since no CTRM layer was applied on the edge areas, where two segments were located, each test on a strengthened slab segment had a non-strengthened reference test.

**Figure 3:**
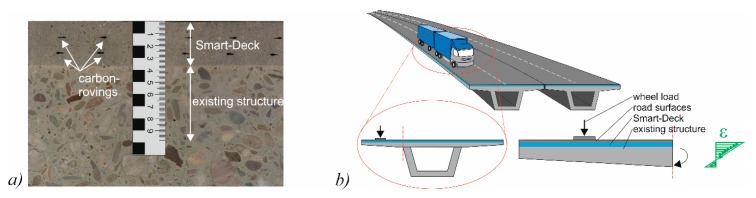
(**a**) Detail of a cut surface; and (**b**) a bridge loaded by a truck and the idealized load setup.

By investigating members with two different steel reinforcement ratios, the varying conditions in existing bridges were represented. Additionally, different failure modes can be expected since the test specimens with low reinforcement ratios fail in flexure while shear failure governs in specimens with higher reinforcement ratios. Along the cut surfaces of the sawn segments, the position of the CFRP grid was examined (Figure 3a). No significant deviation of the intended position was observed.

### 2.3. Investigation of the Strengthening Effect

**Figure 4:**
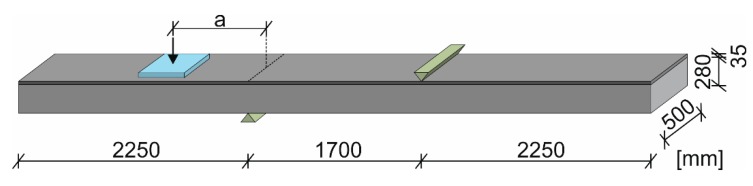
Test setup.

Within the scope of the load bearing tests, a truck located on a bridge’s cantilever was simulated, as shown in Figure 3b. For this purpose, the specimens were simply supported with a distance of 1.70 m between the axles of the supports. The resulting cantilevers of 2.25 m were separately loaded in two partial tests. The support that was more distant from the load was arranged at the topside of the specimen in order to counteract the lifting forces that occurred due to the test setup. The load was induced employing a hydraulic cylinder and a load distribution plate with an area of 40 × 40 cm. These measurements represent the contact surface of a wheel load according to Eurocode 1 [44]. The distance *a* between the center line of the load and the axis of the support according to Figure 4 was 0.7 m, 1.0 m and 1.3 m to investigate the influence of different *a*/*d*-ratios (corresponding to the shear-slenderness) within the shear tests and to vary the level of the bending moment within the flexural tests, respectively. Table 1 gives an overview over the parameters of the test program.

**Figure 5:**
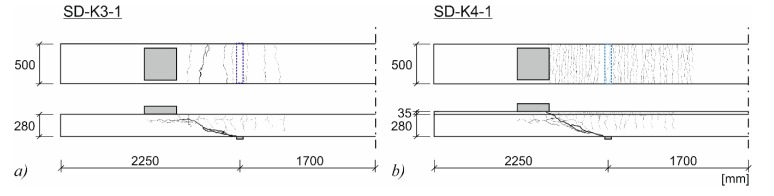
Crack pattern of (**a**) SD-K3-1; and (**b**) SD-K4-1.

All specimens were loaded until failure. Within the shear tests, a characteristic diagonal shear crack occurred (Figure 5). At failure, the cracks opened widely. Regarding the tests on strengthened specimens, a significantly larger number of bending cracks appeared, featuring smaller crack widths compared to the non-strengthened reference tests, like the crack patterns at failure of SD-K3-1 and SD-K4-1, exemplarily shown in Figure 5.

**Figure 6:**
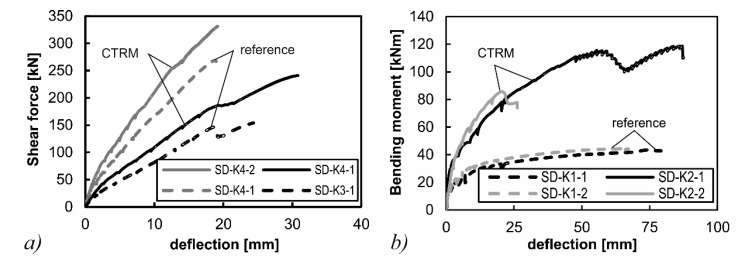
(**a**) Load-deflection-curve of shear tests; and (**b**) the load deflection curve of the bending tests.

Figure 6 shows the load-deformation-curves of the tests. The deflections were measured below the center of the load application. All tests on the strengthened slab segments feature lower deformations compared to their reference tests at the same load level. Within the bending tests on the strengthened specimens, local delamination between the carbon grid and the surrounding mortar occurred. Subsequent modifications of the materials by the project partners aim to prevent this phenomenon. In the shear tests (SD-K3 and SD-K4), the increases in shear capacity were η_V,1_ = 56% and η_V,2_ = 23% (η = (*V*_u,TRC_ − *V*_u,RC_)/*V*_u,RC_), respectively. The increase in flexural capacity within the bending tests was significantly higher. The flexural strengthening rates were η_M,1_ = 174% and η_M,2_ = 91% (η = (*M*_u,CTRM_ − *M*_u,RC_)/*M*_u,RC_), respectively. Table 2 gives an overview of the test results.

These tests show that Smart-Deck, in principle, provides an increased flexural and shear strength of concrete bridge deck slabs. The findings of these experimental investigations and the other experiences made within the scope of the implementation of the entire slab provide important information regarding further development of the materials. Further investigations are in preparation. Other shear tests will be carried out, as well as flexural tests on slab segments with lower reinforcement ratios. Additionally, one shear and one bending test under cyclic loading are currently planned in order to investigate the load-bearing behavior of the strengthened member under fatigue loading.

## 3. Strengthening of Webs with a CTRM layer

### 3.1. Concept and Preliminary Investigations

**Figure 7:**
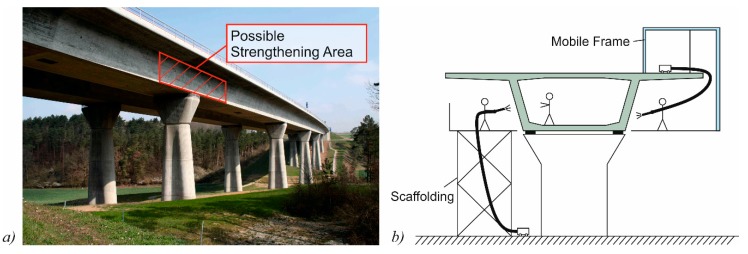
(**a**) Possible are of CTRM strengthening (image edited by the authors, “A71-Thalwassertalbruecke”, author: Störfix, licensed under CC BY-SA 3.0); and (**b**) the application method.

The possibility of strengthening the webs of pre-stressed concrete bridges was investigated in cyclic and static shear tests on pre-stressed concrete beams [40,41]. This method allows for a local strengthening of critical areas, mostly in the vicinity of bridge columns instead of the whole structure (Figure 7a). The main advantage of local strengthening is the reduced effort, and especially the reduced dead load that is added to the structure. For experimental validation, pre-stressed concrete beams with an I-shaped cross-section were strengthened on the webs using a CTRM layer. The CTRM layer consists of a carbon fiber grid in combination with a sprayed mortar. In its load-bearing behavior, an I-shaped cross-section resembles common pre-stressed box girder bridges (Figure 7b), for which this strengthening method could be utilized. In the test specimens, an elaborate anchoring of the strengthening layer in the top and bottom chord was omitted. According to the truss analogy, a strengthening-layer without anchorage in the chords should have little to no effect on the ultimate shear strength. Nevertheless, a strengthening effect of a grid reinforced layer can be expected according to yield line theory [45], especially due to the rovings in the horizontal direction.

**Figure 8:**
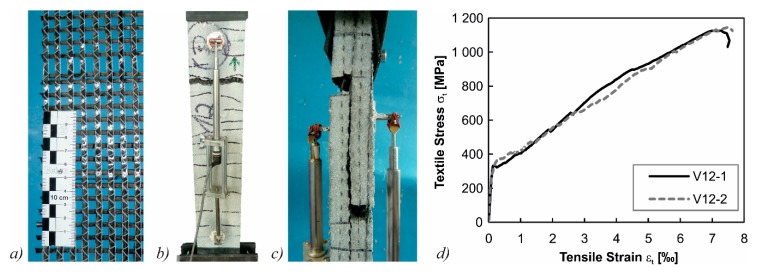
(**a**) Unimpregnated carbon textile; (**b**) tensile test setup; (**c**) pull-out of rovings after failure; and (**d**) stress-strain relationships of tensile tests with an un-impregnated carbon grid.

In a first step, an adequate combination of sprayed mortar and textile material for the strengthening of the beams had to be found. For this, tensile tests similar to [46] were conducted on specimens with dimensions of 100 mm width, 880 mm length, and a thickness of 25 to 30 mm (Figure 8b,c). Combinations of two different matrices and one to four layers of textile grid (alkali-resistant glass/carbon impregnated with epoxy resin/styrene-butadiene/un-impregnated carbon grid) were investigated. In Table 3, the different mean values of the maximum tensile stresses in the textile for different combinations of parameters are summarized. In the end, polymer-modified dry-spray mortar (SPCC) with a maximum aggregate size of *d*_ag_ = 2 mm and an unimpregnated carbon grid with an area of *a*_tex_ = 55 mm²/m was used as the textile reinforcement material (Figure 8a). The mean tensile strength of the carbon grid in these tests was σ_t_ = 1136 MPa (Figure 8d).

### 3.2. Test Specimens and Test Setup

**Figure 9:**
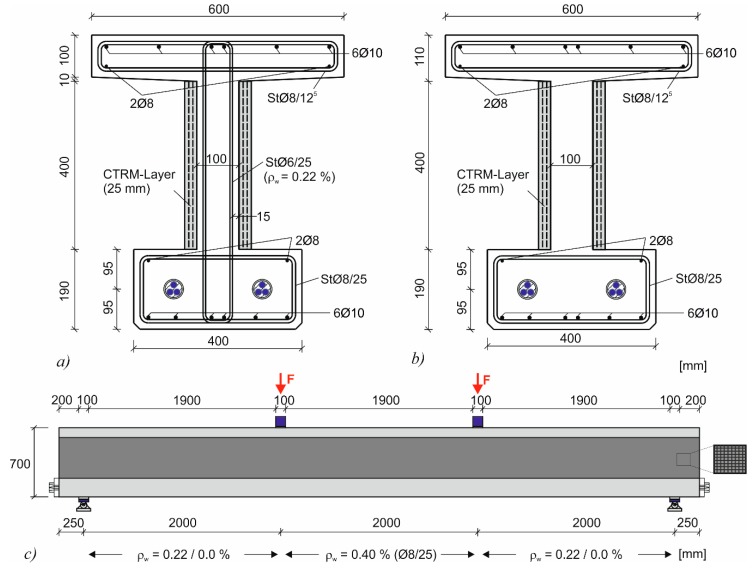
(**a**) Strengthened cross-section with shear reinforcement (CTRM-M-22-7); (**b**) the strengthened cross-section without shear reinforcement (CTRM-I-O-5); and (**c**) the longitudinal system.

The experimental results of the strengthened test beams were compared to similar test beams from previous research projects without a CTRM layer which served as a reference [47,48]. The cross-section of the test beams had a total width of 0.6 m and a web width of 0.1 m (Figure 9a,b). The beams had a total length of 6.5 m and cross-section height of 0.7 m (Figure 9c). The point loads were located in the third points of the beam with a resulting shear slenderness of *a*/*d* = 3.3. The tests were performed on a member with a low amount of shear reinforcement (ρ_w_ = 0.22%) which was strengthened with CTRM (CTRM-M-22-7). This member was compared to identical members without strengthening (M-22-7 and M-22-3) from a previous project [48]. Another member without shear reinforcement with CTRM-strengthening was produced (CTRM-I-O-5) which was also previously tested without strengthening (I-O-5) [47]. The test beams were subjected to 1.2 to 3.1 million load cycles using different peak and valley loads. In difference to tests by other authors [42], the strengthening layer was not anchored the compression or tension chord.

### 3.3. Material Properties

Normal strength concrete with a maximum aggregate size of 8 mm was used. In Table 4 the mechanical concrete and shotcrete properties at the time of testing of the specimens are given. The number of test specimens is given in brackets. The cylinder strength *f*_cm,cyl_ and the splitting tensile strength *f*_ct,split_ were determined on cylinders with *h* = 300 mm and a diameter of *d* = 150 mm. The cube strength *f*_cm,cube_ was determined on cubes with an edge length of 150 mm. The axial tensile strength *f*_ct,ax_ was determined on drilled cores with *h* = 90 mm and *d* = 45 mm that were either drilled from the web of the beams or a flexural tensile test specimen. The mechanical properties of the shotcrete were determined on prisms with a length of 160 mm and a width and height of 40 mm. After testing the flexural tensile strength *f*_ct,flex_, the compressive strength *f*_cm,prism_ was determined from the remaining prismatic samples.

The mild steel reinforcement in each beam consisted of normal strength steel bars (*f*_yk_ = 500 MPa). The mechanical properties of the shear reinforcement are given in Table 5. The beams were pre-stressed using two tendons, each consisting of three 0.6” (15.2 mm) strands of pre-stressing steel St1570/1770 with a cross-sectional area of 3 × 140 mm. The pre-stressing forces at the time of testing and the mechanical properties of the tendons are given in Table 6.

### 3.4. Test Specimens and Test Setup

**Figure 10:**
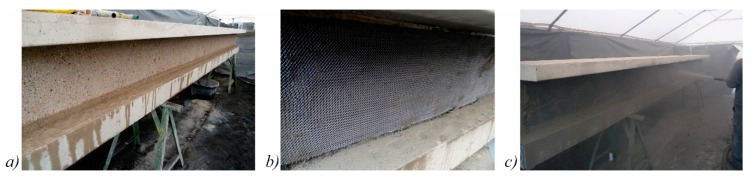
(**a**) Test specimen after shot blasting; (**b**) the application of the textile reinforcement; and (**c**) the application of shotcrete layers.

The test specimens were strengthened about three weeks after pre-stressing. In the first step, the surface of the webs was roughened by sandblasting. Since the sand patch method cannot be applied to vertical interfaces to determine the roughness, an equivalent procedure with gypsum based on the cement paste method was used [49]. Accordingly, the surface roughness *R*_t_ ranged from 1.1 to 2.4 mm. Prior to the application of the strengthening layer, the surface of the webs was cleaned and moistened (Figure 10a). The CTRM was applied layer by layer with three layers of shotcrete and two layers of carbon grid on each web (Figure 10b). The total thickness of the strengthening layer amounted to 25 mm. After strengthening, the shotcrete was moistened for another three days to ensure a sufficient hydration.

### 3.5. Test Results

#### 3.5.1. Load Regime

The amplitudes of the loads and the number of load cycles are summarized in Table 7. For the beams without shear reinforcement, a peak load of about 75% of the shear crack load was applied. The beams with shear reinforcement were loaded until shear cracking occurred so that the stirrups were activated. The highest load *V*_max_ (HL) was then chosen at 110% of the shear crack load to activate the stirrups and was increased if no significant damage occurred after 10^6^ load cycles. The deflection of the test specimens was measured beneath the loading points by displacement transducers. A digital image correlation system was used to measure the shear crack growth dependent of the load cycles.

#### 3.5.2. Specimen CTRM-I-O-5

**Figure 11:**
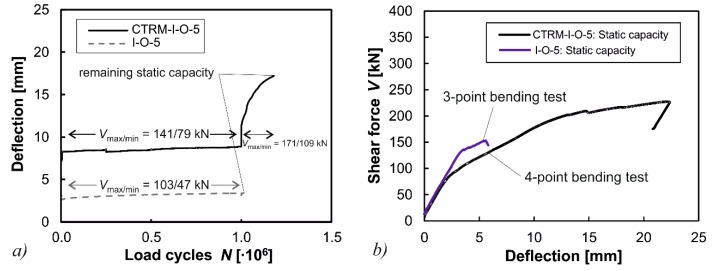
(**a**) Comparison of vertical deflections under cyclic loading for tests without shear reinforcement; and (**b**) the comparison of the remaining shear capacities of non-strengthened and strengthened specimens.

For the test beams without shear reinforcement (I-O-5 and CTRM-I-O-5) the highest load of the strengthened beam CTRM-I-O-5 was increased by 40% in comparison to the non-strengthened beam I-O-5. Nevertheless, the beam strengthened with CTRM did not show any signs of fatigue failure. In the following step, the loading was increased further so that the maximum load was almost equal to the initial shear crack load of the non-strengthened specimen I-O-5. At this level, the non-strengthened beam would have failed immediately, whereas the beam with CTRM sustained another 180,000 load cycles despite the formation of a large shear crack (Figure 11a). Although additional load cycles would have been possible, the testing was aborted at this point due to large deflections. The remaining static capacity of the specimen CTRM-I-O-5 was *V*_ult_ = 233 kN, whereas the original specimen without CTRM had a remaining capacity of only *V*_ult_ = 158 kN (Figure 11b).

#### 3.5.3. Specimen CTRM-M-22-7

**Figure 12:**
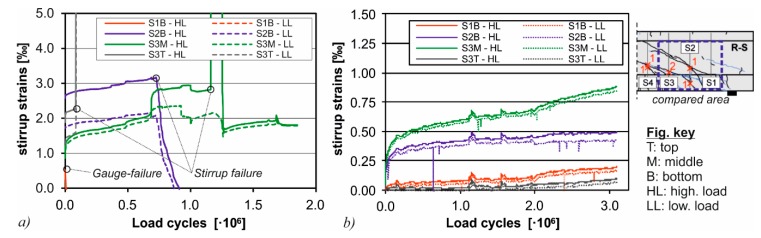
Stirrup strains in the vicinity of the supports for (**a**) M-22-7; and (**b**) CTRM-M-22-7.

The stirrup strains of the specimens with shear reinforcement, which were measured by strain gauges, are shown in Figure 12 for specimens M-22-7 (without CTRM-strengthening) and CTRM-M-22-7. The peak load of the specimen CTRM-M-22-7 was increased by about 30% compared to the previous specimen M-22-7. In the previous experiment without CTRM-strengthening, various stirrups failed during the first 10^6^ load cycles which can be seen from the progression of the curve in Figure 12a. In contrast, the beam strengthened with CTRM did not exhibit any damage on the stirrups after 2 × 10^6^ load cycles, after which the amplitude was increased further (Figure 12b). After the increase of the amplitude, some stirrups failed and the deflection grew moderately. Even then, the beam was able to sustain another 1.1 × 10^6^ load cycles after which the test was aborted. This behavior indicates a considerable load transfer over the CTRM strengthening, relieving the existing stirrups.

**Figure 13:**
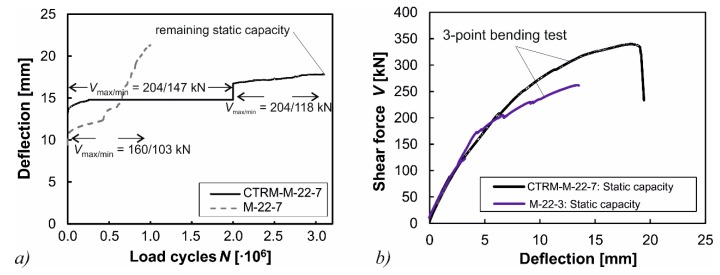
(**a**) Comparison of vertical deflections under cyclic loading for tests with shear reinforcement; and (**b**) the comparison of the remaining shear capacities of the non-strengthened and strengthened specimens.

The deflections in dependence of the load cycles and the load-deflection curves under static loading are illustrated in Figure 13. The strong increase of the deflection of the non-strengthened beam M-22-7 indicates a progressive failure of stirrups during the first 10^6^ load cycles (Figure 13a). On the other side, the strengthened specimen CTRM-M-22-7 did not show any signs of a progressive fatigue failure even after increasing the amplitude after 2.0 × 10^6^ load cycles. The remaining capacity of the beam CTRM-M-22-7 amounted to *V*_ult_ = 350 kN (Figure 13b). The remaining capacity of the original beam M-22-7 was not determined due to its considerable damage in the stirrups. However, another previous test beam M-22-3 with the same pre-stressing, but subjected to smaller highest loads, had a remaining capacity of *V*_ult_ = 264 kN. It can, therefore, be seen that the CTRM strengthening had a considerable effect on the remaining shear capacity for the beams with shear reinforcement as well.

**Figure 14:**
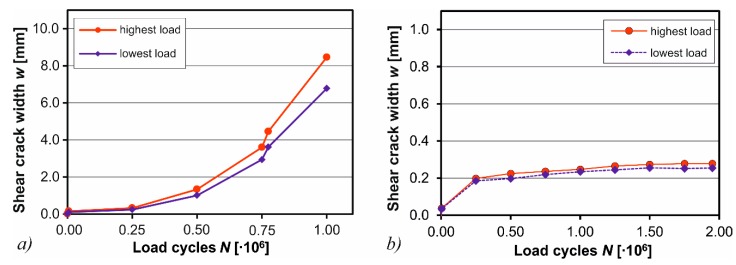
Shear crack widths *w* according to digital image correlation for (**a**) M-22-7 and (**b**) CTRM-M-22-7.

This is also indicated by the crack width measurements in the shear span made by digital image correlation (DIC). The DIC technique is frequently used in shear tests to accurately analyze the crack growth [50,51]. Here, the Aramis system (v5.4, GOM GmbH, Braunschweig, Germany) was used [52]. The shear crack widths for specimen M-22-7 dependent of the load cycles are illustrated in Figure 14a for highest and lowest loads. As can be seen, the shear crack widths increase rapidly to over 8 mm during the first 10^6^ load cycles in correspondence with the failure of stirrups according to Figure 12a and the increase of deflections according to Figure 13a. For the strengthened specimen CTRM-M-22-7, the crack widths were measured during the first 2 × 10^6^ load cycles (Figure 14b). The diagram, which is scaled down by one order of magnitude compared to Figure 14a, shows that the shear crack widths are considerably smaller ranging from 0.2 to 0.3 mm. Additionally, the crack widths do not increase exponentially as for the non-strengthened specimen, which illustrates the stabilizing effect of the CTRM-strengthening.

#### 3.5.4. Summary

The strengthening effect of textile-reinforced sprayed mortar on the webs of pre-stressed concrete beams was tested on two specimens under cyclic and fatigue loading. The test results were compared to similar beam tests without CTRM strengthening. In summary, the following conclusions can be drawn from the tests:

- Although the strengthening layer was not anchored in the compression or tension chord, a significant strengthening effect was observed. This effect can be explained by the contribution of the horizontal rovings which are activated at crack opening.
- For the specimen without shear reinforcement, additional 180,000 load cycles could be sustained after shear crack formation which results in a much more ductile behavior in comparison to non-strengthened specimens.
- For the specimen with shear reinforcement a significant reduction of stirrup strains was observed, as well as significantly smaller shear crack widths. By this, a progressive fatigue failure was prevented by the CTRM-strengthening.
- A bond failure between old concrete and strengthening layer could not be observed in any of the tests as the surface was sufficiently roughened and cleaned prior to strengthening. However, if the surface is not prepared according to the applicable standards [49], bond failure might occur, neutralizing a potential strengthening effect.

These tests have therefore shown, that there was a considerable increase of the fatigue and ultimate capacity of the strengthened beams, although the CTRM layer was not anchored in the compression or tension chord. Although, a general design method to predict the strength of members with a retroactive CTRM-strengthening cannot be derived based on these tests, the investigations indicate that a further investigation of this strengthening method might be quite promising for practical applications.

## 4. Conclusions

The use of carbon textile-reinforced mortar (CTRM) offers an innovative alternative for strengthening measures by combining the advantages of light, glued CFRP strips and the better bond characteristics of an additional concrete layer. Different fields of application were investigated and described in the paper: a considerable increase of the flexural strength and shear strength due to the CTRM-strengthening in the tension zone of bridge deck slabs was observed. Apart from the strengthening function, the CTRM layer can be used for real-time humidity monitoring and a preventive cathodic corrosion protection. These features potentially enable a significantly longer service life of the infrastructure due to the extended corrosion protection which is necessary, especially for the bridge deck surface.

A significant increase of the shear strength under static and cyclic loads was also obtained by strengthening the web of I-shaped profiles with a CTRM layer. Although the strengthening layer was not anchored in the chords of the test beams, the shear crack widths were reduced by a factor of 30 relieving the stirrups and, thus, enabling a very ductile behavior of the member. Within the scope of further experimental investigations, the strengthening method is to be optimized for use in practical applications. In addition to experimental investigations, theoretical investigations are required regarding practicable design models to realistically predict the strength of beams and slabs strengthened with CTRM.

## Figures and Tables

**Table 1:** Amount of CTRM and steel, effective depth *d*_s_ of the RC slab and the load distance *a*.

Specimen	CTRM (mm²/m)	Rebar (cm²/m)	*d*_s_ (m)	*a* (m)
SD-K1-1	0	5.24	0.21	1.3
SD-K1-2	0	5.24	0.21	1.0
SD-K2-1	280	5.24	0.215	1.3
SD-K2-2	280	5.24	0.205	1.0
SD-K3-1	0	25.13	0.215	1.0
SD-K3-2	0	25.13	0.205	0.7
SD-K4-1	280	25.13	0.235	1.0
SD-K4-2	280	25.13	0.22	0.7

**Table 2:** Maximum bending/shear capacity *M*_max_/*V*_max_, maximum deflection *w*_max_, and strengthening degree η.

Specimen	CTRM (mm²/m)	*w*_max_ (mm)	*M*_max_ (kNm)	*V*_max_ (Kn)	η (%)
SD-K1-1	0	89	43.4	-	-
SD-K1-2	0	71	44.9	-	-
SD-K2-1	280	73	118.7	-	174
SD-K2-2	280	22	85.7	-	91
SD-K3-1	0	26	-	154	-
SD-K3-2	0	32	-	268.5	-
SD-K4-1	280	24	-	240.9	56
SD-K4-2	280	22	-	331.5	23

**Table 3:** Mean values of maximum textile stresses in the tensile tests for different textile materials and sprayed mortars.

Textile	Shotcrete; *d*_ag_ = 4 mm	SPCC; *d*_ag_ = 2 mm
carbon fiber + epoxy resin	2397 MPa	2928 MPa
AR-glass fiber + epoxy resin	1640 MPa	2076 MPa
carbon + styrol-butadien (type 1)	935 MPa	1198 MPa
carbon + styrol-butadien (type 2)	362 MPa	276 MPa
unimpregnated carbon	-	1136 MPa

**Table 4:** Mechanical properties of the concrete used in beams and the spray mortar.

Specimen	Concrete					Shot Mortar (SPCC)	
	*f*_cm,cyl_ (MPa)	*f*_cm,cube_ (MPa)	*f*_ct,ax_ (MPa)	*f*_ct,split_ (MPa)	*E*_cm_ (MPa)	*f*_cm,prism_ (MPa)	*f*_ct,flex_ (MPa)
I-O-5	29.4 (6)	34.9 (10)	2.80 (10)	2.54 (3)	22,200 (6)	-	-
CTRM-I-O-5	42.3 (6)	47.0 (6)	2.98 (13)	3.44 (6)	26,790 (6)	53.8 (10)	6.30 (5)
M-22-3	35.3 (6)	38.6 (9)	2.68 (15)	2.43 (4)	24,833 (6)	-	-
M-22-7	32.0 (7)	35.4 (9)	2.55 (15)	2.59 (5)	23,900 (7)	-	-
CTRM-M-22-7	43.0 (6)	47.2 (4)	3.10 (14)	3.25 (6)	25,140 (6)	44.6 (8)	7.12 (4)

**Table 5:** Mechanical properties of the shear reinforcement.

Specimen	*f*_y;0,2_ (MPa)	*f*_t_ (MPa)	*E*_s_ (MPa)
M-22-3	587	626	200,777
M-22-7	587	626	200,777
CTRM-M-22-7	595	633	203,800

**Table 6:** Pre-stressing values and properties of tendons.

Specimen	*P*_mt_ (kN)	σ_cp,mt_ (MPa)	*f*_p0,2_ (MPa)	*f*_pt_ (MPa)	*E*_p_ (MPa)
I-O-5	320	1.78	1764	1950	190,000
CTRM-I-O-5	327	1.82	1764	1950	190,000
M-22-3	320	1.78	1764	1950	190,000
M-22-7	314	1.75	1764	1950	190,000
CTRM-M-22-7	329	1.83	1764	1950	190,000

**Table 7:** Number of load cycles and amplitudes.

Specimen	*V*_crack_ (kN)	Load Cycles × 10³		*V*_max_ (kN)	*V*_min_ (kN)	ΔV (kN)
		N_i_	ΣN_i_			
I-O-5	176	1000	1000	103	47	56
		1011	2011	102	35	67
CTRM-I-O-5	188	1000	1000	141	79	62
		180	1180	171	109	62
M-22-7	145	1853	1853	160	103	57
CTRM-M-22-7	185	2000	2000	204	147	60
		1100	3100	204	118	86
